# Preaching Through the Choir. What Interprofessional Education Can Learn From Choir Singing

**DOI:** 10.5334/pme.1182

**Published:** 2024-05-02

**Authors:** Juliëtte Anna Beuken, Felicitas Biwer

**Affiliations:** 1 Department of Educational Development and Research/School of Health Professions Education (SHE), Faculty of Health Medicine and Life Sciences, Maastricht University, The Netherlands

## Abstract

Collaboration between healthcare professionals from different backgrounds is a true art to be mastered. During interprofessional education (IPE), learners from different professions learn with, from and about each other. Landscape of Practice (LoP) theory can offer insight into social learning in IPE, but its application is rather complex. We argue that choir singing offers a helpful metaphor to understand different concepts in LoP (*brokers, engagement, imagination* and *alignment*) and how they are manifested in IPE. Based on similarities between choir singing and IPE, we present four lessons: 1) *The teacher sets the tone: a lesson for brokers;* 2) *You can only learn so much alone: a lesson for engagement;* 3) *Listening is not as easy as it sounds: a lesson for imagination* and 4) *A song is more than the sum of its parts: a lesson for alignment*. Moreover, we reflect on differences between choir singing and IPE, and insights from these differences.

## Introduction

Healthcare inherently requires collaboration between healthcare professionals from different backgrounds, who understand and appreciate each other’s contributions to care. Interprofessional education (IPE) prepares learners in health professions education (HPE) to work together in complex healthcare systems. IPE is organized in different contexts and scales, e.g. student-run clinics [1] or online IPE in times of crisis [2], but is always a social learning practice. Learners in IPE learn with, from and about each other in communities of practice (CoPs) and Landscapes of Practice (LoPs). CoPs are “*groups of people who share a concern or a passion for something they do and learn how to do it better as they interact regularly*” [3]. In LoPs learners navigate within and through different CoPs [4]. Successfully engaging in a LoP requires learners to understand their own and the others’ identity. The boundaries in LoPs, where different communities meet, are especially fruitful for learning [5]. Sometimes *brokers*, who are knowledgeable about the different CoPs and their connections, are present at the borders of the LoP. These brokers may facilitate the transfer of knowledge between communities [6], by stimulating three processes of identification: engagement, imagination and alignment. As explained by de Nooijer, Dolmans and Stalmeijer [7], at the boundaries learners learn how they are or will be relevant to one another (*engagement*), get a sense of their own and others’ professional context and how they relate (*imagination*), and how their activities can or will align in collaborations (*alignment*). To the authors of this paper (two early-career teachers and researchers in HPE) the different concepts of LoP and how they apply to IPE are rather complex. Yet, especially for new teachers in HPE, LoP is an important concept to grasp in order to support learners in developing their professional identity and engaging successfully in collaboration in education and the future work field.

When things become complex, the authors find relaxation in choir singing. Every week, we get together with over fifty people from different backgrounds and with different voices, who have one thing in common: they enjoy singing together. But bringing people together who love to sing does not yet make a choir. Many things need to happen between the first and final rehearsal, in order to become a strong choir and ultimately perform a (good) concert.

With some imagination, we can see overlap between singers that want to form a choir and perform a good concert, and learners in (undergraduate all the way to continuing) HPE that need to learn to collaborate and provide good healthcare. In the former, rehearsals are in place to make sure that singers learn their different parts *and* learn to sing them together. In the latter, IPE prepares learners for their profession *and* to collaborate with other professions. We argue that both members of the choir as well as learners in IPE navigate in a landscape of practice, in which they go through processes of engagement, imagination and alignment. Assisted by knowledgeable brokers, they can learn with, from and about each other. Exploiting the power of this overlap between choir singing and IPE, we explore similarities between the two that could provide practical insights for common challenges in IPE.

## Basics of Choir Singing

Typically, a choir has four voice groups, from high to low: sopranos, altos, tenors and basses. Although levels of experience in singing may differ, a key requirement is that singers can keep their tone. The four voice groups all sing different parts, often captured in sheet music. Most choirs have a conductor who is familiar with all different parts (though not necessarily able to sing all) and has a vision for what a song should sound like and how the singers should sing together to make music. In our choir singing metaphor for IPE, the singers are learners, the conductor is a teacher, the different voice groups are different professions, and the choir is a landscape of practice.

## Similarities Between Choir Singing and IPE

In four lessons, we link choir singing to landscapes of practice concepts and IPE.

### The teacher sets the tone: a lesson for brokers

In choir singing, the conductor takes leadership as the singers practice songs. The conductor knows how the different parts should be sung, how they relate to each other and sound together, and conveys this knowledge by practicing parts separately and together. While there are limitations to their abilities to mimic each part, conductors are equipped to transfer knowledge by using an instrument for notes outside their reach. For example, our conductor is a bass-singer. When he demonstrates the parts of soprano-singers, he will sing as much as possible with a lower voice, and whenever he cannot reach a note, he uses the piano instead. The conductor’s understanding of all parts in a song is crucial in transforming our separate voices to a choir sound. Moreover, experienced singers will observe challenges in their own and other voice groups from their position within the choir. When these observations are shared with and recognized by the conductor, experienced singers thus also contribute to the choir sound.

In LoP, *brokers* (conductor/teacher) act at the boundaries to facilitate the exchange of knowledge. They can contribute to this exchange even if they do not strongly identify with one or both sides of the boundary [6]. For brokers to share their knowledge, their peripheral participation in different communities (voice groups/professions) needs to be recognized by those within the community.

In IPE, formal and informal teachers may serve as brokers on the borders of professional communities. They may not know everything about the professions of the learners they teach, but they are *knowledgeable*. They should have a basic understanding of the roles of different professions and the connections between them. The role of brokers in IPE is challenging, for example due to a lack of confidence in cross-profession knowledge among IPE teachers [8]. It is thus important to empower brokers and to establish trust between learners and teachers, for example by supporting interprofessional teacher teams. Moreover, teachers will not always be present at interprofessional encounters or may not be recognized as brokers, especially in workplace-based learning. Learning from choir singing, formal (conductor) and informal (experienced singers) brokers are crucial in IPE as they can demonstrate connections between professions and be a role model for collaboration. Teachers who act as brokers in IPE need to be recognized as such and be supported to build competence and trust in their knowledgeability.

### You can only learn so much alone: a lesson for engagement

In choir singing, most singers will practice their parts at home, using recordings or instruments to learn their part. Such solo practice is helpful when a singer is insecure or struggles to sing their part in combination with other parts. However, solo practice cannot replace choir practice. Most of the music is learned during rehearsals, with singers from different voice groups. During choir practice, singers learn what their own part sounds like, how it relates to the other parts and how their part is relevant to the whole song. By rehearsing regularly with each other, singers notice challenges in the song, which stimulates further choir- or solo practice. When a new choir project starts, singers bring their experiences from other choirs or past projects with them. They need to invest time to get to know the strengths and weaknesses of members of their own and the other voice groups but can also bring new ideas for productive choir practice with them (e.g. a new warming-up exercise). Finally, when we sing together, we hear what makes a song beautiful, the harmonies and dissonances. More than once, the first time we hear different voices come together results in goose bumps.

In LoP, *engagement* describes an understanding of how different professions are relevant to one another. This requires learners to know themselves and the other professions, to understand perspectives and actions of different professions in different situations [7], and ultimately to recognize the strength of combined forces. Over the course of their careers, people move through different communities of practice and take their experiences to other contexts. The practice of engagement describes understanding the relevance to one another and others’ expertise and learning new skills and perspectives from each other. To wonder and to improve are essential first steps in collaboration.

In IPE, learners get opportunities to engage with learners from other professions. Whatever they learned about their own profession in their education silo gets put into the context of their joint effort of care [9]. Breaking down professional silos may be confusing, and there is ample discussion about when IPE should be introduced in HPE. Learning from choir singing, we can conclude that learning mostly takes place when different professions meet regularly and early on, even when learners are still forming their own identity. It allows learners to see their own and other professions’ challenges and how to tackle these over time. Monoprofessional learning can surely be supportive, but never central, in learning to collaborate.

### Listening is not as easy as it sounds: a lesson for imagination

In choir singing, listening to what others do as you sing your own part is not as easy as it sounds, and yet crucial. Being able to sing one song with different parts takes understanding of how these different parts relate to one another. The conductor has a role in forming this understanding, but ultimately it takes *listening* to other voice groups practicing their part and *singing* the song together. For example, as one voice group practices their part, the conductor can encourage the others to practice their part too, in their head or by humming along. Practicing songs in big circles, standing next to singers of other voice groups, singers can hear the harmonies and dissonances between their own and other voice groups, and become more aware of their own part, understanding challenges in the music for different voice groups.

In LoP, *imagination* refers to how we see our own role and responsibilities and how we see ourselves in relation to each other [7]. Interacting with learners from different professions, learners move towards the boundaries of their respective communities. This way, they see their role in the future professional context and how this relates to others’ roles. Healthcare professionals from different backgrounds actively listen to each other to learn both their own and others’ responsibilities in healthcare. Imagination requires authentic situations in which learners can experience these professional relations. Ideally, learners from different professions can discuss the dynamics in these relations with their peers and teachers.

In IPE, learners need to have opportunities to experience and reflect on their roles and responsibilities in relation to other professions and how they matter to one another. Authentic interprofessional situations allow learners to imagine their (future) relations to one another. For example, in an outpatient psychiatry rotation, a physician in training might observe someone from a different profession (e.g. a community nurse), to learn about their professional roles and (possible) relation to one another. Yet, mere observations would not suffice; this needs to be complemented with intra- and interpersonal reflections. Learners could discuss what certain situations would mean for both professions, and how they could collaborate to optimize care. This way, observing other professions during rotations might help learners to understand and respect the roles of other professions and provide learners with a more global perspective on healthcare [10]. Learning from choir singing, imagination mostly takes place when learners from different professions are exposed to each other in authentic interprofessional situations and guided in inter- and intrapersonal reflection on their roles and relations. Learners need to be open to being vulnerable (like singing alone, without singers from their own voice group next to them) to notice common benefits and challenges of interprofessional collaboration.

### A song is more than the sum of its parts: a lesson for alignment

In choir singing, it is important to make sure that different voices align. When a new choir project starts, singers with different choral experiences come together. Some might be able to sight-read music; others might need more time and support from the conductor to learn their parts. To work together in a new project, singers need to align their expectations and attitudes to another. For example, if singers of one voice group sing their part so loud that other voice groups are barely audible, colorful harmonies will disappear and the goal of making music together is lost. Therefore, singers aim to stick to formal agreements in the score and receive instructions from the conductor. But most importantly, singers become aware of each other’s voices. As they start to sing together, the importance of each voice group becomes abundantly clear. This emphasizes how important it is to support each other, and to align the singing. Only then, a group of singers *becomes* a choir.

In LoP, alignment describes the process of coordinating actions as professionals collaborate in the context of a healthcare system to provide care. This process requires professionals to feel free to speak up and ask questions, and show and feel respect from and for other perspectives [7]. When moving from one LoP to another, people bring their experiences and baggage, which can enrich but also burden collaboration if it is not communicated.

In IPE, learners need to practice together to see how the different professions align with one another. Learning from choir singing, an IPE program that offers opportunities to practice collaboration with peers from other professions (other voice groups or other choirs) in the context of the healthcare system (the performing choir), would allow learners to understand and ultimately *adapt* to activities in this system. To overcome intergroup tensions [11], IPE should stimulate learners to form connections outside of their respective professional groups (recognize the role of other voice groups), and nurture a shared group identity (forming a choir). This way, (future) healthcare professionals learn to tackle problems together rather than pointing fingers, and ultimately work as one.

## Differences between Choir Singing and IPE

Now that we have explored similarities between choir singing and IPE, it is important to also note some differences. These are both side notes to the comparison we draw in this paper, and the reason that this comparison provides interesting insights.

First, IPE is oftentimes challenged by logistics. When we bring learners from different professions together, we need to adjust to different schedules and accreditation systems, while choir singers meet at a fixed time and place (though personal logistical challenges might still occur). Second, due to curricular variety, we work with many different teachers, and leadership in IPE shifts. Different groups come together with different teachers or even no identified teacher present. Contrarily, choir singers are always led by the same teacher: the conductor. Third, choir singers have an ever-obvious shared goal; rehearse and perform songs as well as possible. As explained before, choir singers inevitably know that this goal cannot be achieved if only one voice group excels. The shared goal of healthcare professionals may not be as visible throughout HPE. Where healthcare professions are oftentimes subject to hierarchy [12], the different voice groups in choirs are respected to be equally important to the shared goal. Lastly, as healthcare professions progress in HPE, their professional identity tends to become stronger than their identification with the healthcare workforce in general. The counter-process of interprofessional identity formation is gaining interest in IPE and HPE [13]. Choir singers identify only to some extent with their voice group and much more with ‘their’ choir (e.g., we have choir merchandise).

Learning from these differences, we consider another four lessons for IPE: First, redesign or align the different professional training systems to create dedicated shared spaces for IPE. Second, consider the role of more permanent teachers, who, while not present for all IPE experiences or part of interprofessional teams, can continuously serve to help learners recognize and reflect on interprofessional interactions and relations. Third, increase emphasis that the goal of quality patient care is one shared across professions, and all professions should be respected as equally important for reaching this goal. Lastly, increase efforts and research into how to simultaneously develop professional and interprofessional identities.

## Final Remarks

As showcased above, there are differences as well as similarities between choir singing and IPE. Lessons can be learned from the ways in which singers in a choir learn together from the first rehearsal to the final concert. This inherently collaborative process ultimately strengthens a sense of belonging and togetherness amongst its singers [14]. We can only hope that IPE does this for healthcare professionals, too.
